# A Simple and Efficient Deep Learning-Based Framework for Automatic Fruit Recognition

**DOI:** 10.1155/2022/6538117

**Published:** 2022-02-21

**Authors:** Dostdar Hussain, Israr Hussain, Muhammad Ismail, Amerah Alabrah, Syed Sajid Ullah, Hayat Mansoor Alaghbari

**Affiliations:** ^1^Department of Computer Sciences, Karakoram International University, Gilgit 15100, Pakistan; ^2^Department of Information Systems, College of Computer and Information Sciences, King Saud University, Riyadh 11543, Saudi Arabia; ^3^Department of Information and Communication Technology, University of Agder, Kristiansand, Norway; ^4^Botany Department, Faculty of Science, Taiz University, Taiz 6803, Yemen

## Abstract

Accurate detection and recognition of various kinds of fruits and vegetables by using the artificial intelligence (AI) approach always remain a challenging task due to similarity between various types of fruits and challenging environments such as lighting and background variations. Therefore, developing and exploring an expert system for automatic fruits' recognition is getting more and more important after many successful approaches; however, this technology is still far from being mature. The deep learning-based models have emerged as state-of-the-art techniques for image segmentation and classification and have a lot of promise in challenging domains such as agriculture, where they can deal with the large variability in data better than classical computer vision methods. In this study, we proposed a deep learning-based framework to detect and recognize fruits and vegetables automatically with difficult real-world scenarios. The proposed method might be helpful for the fruit sellers to identify and differentiate various kinds of fruits and vegetables that have similarities. The proposed method has applied deep convolutional neural network (DCNN) to the undertakings of distinguishing natural fruit images of the Gilgit-Baltistan (GB) region as this area is famous for fruits' production in Pakistan as well as in the world. The experimental outcomes demonstrate that the suggested deep learning algorithm has the effective capability of automatically recognizing the fruit with high accuracy of 96%. This high accuracy exhibits that the proposed approach can meet world application requirements.

## 1. Introduction

We are in an era where we still use bar code technology in fruit shops and supermarkets to get fruit prices and to get other information such as source traceback. This is a big challenge for shopkeepers to remember and manage the bar codes for individual fruit categories. Machine learning-based algorithms achieved significant attention in object detection and recognition [1]. Fruit shops and supermarkets pack fruit and vegetables inside the small boxes and then use bar codes to determine their prices. However, most of the customers prefer to pick their fruits rather than prepackaged ones. In the history of the fruit recognition system for malls, Bolle et al. [2] were the researchers who invented a straightforward fruit recognition system comprising of a join scale and image system. The convolutional neural network (CNN) is a neural network that can be used to enable machines to visualize things and perform a task such as an image classification and recognition [3]. The CNN can take input images, process them, and classify certain classifications. Image processing uses CNNs as one of the most common deep learning techniques [4]. Nuske et al. [5] proposed a visual grape detection approach to yield estimation in vineyards. The authors used both visual texture and shape for berry detection. The approach calibrated berry count for yielding the individual vineyard rows and to predict within 9.8% of weight from the actual crop. Everingham et al. [6] provided a review of the visual object classes (VOC) challenge from 2008–2012. They introduced some algorithms on the datasets of VOC along with evaluation metrics to analyze their performance. Song et al. [7] proposed an automatic method that takes multiple images for recognizing and counting the fruit of varying colors and complex shapes. Zitnick and Dollár [8] proposed a simple-box-object score to measure the number of existing edges in the box minus members and overlapped edges in the boundary of that box. Kapach et al. [9] presented a broad review of state-of-the-art solutions used in machine vision for harvesting robots. Yamamoto et al. [10] developed a method using a conventional digital RGB camera along with machine learning to detect correctly different fruits of intact tomato, including immature, mature, and young fruits. The fruit detection results of this method on test images gave a recall of 0.80 and a precision of 0.88. Girshick [11] proposed a fast region-based convolutional neural network (Fast R-CNN) method for object detection. The fast R-CNN can train the deep VGG-16 network nine times faster than R-CNN. Wang et al. [12] established a computer vision system for rapid, automated, and accurate detecting and registering apples from sequential input images. To reduce the natural illumination variance, it works with artificial controlled lighting at night time. Some of the prior works [13, 14] have discussed the problems and challenges in fruit recognition systems and have also proposed deep learning-based algorithms for fruit recognition. Mia et al. [15] performed a computer vision approach in-depth exploration to recognize the rare local Bangladesh fruits. These local fruits are classified using the features extracted from captured images. Some of the prior studies [16–18] use a transfer learning-based approach for fruit recognition tasks to lower the number of parameters and the cost of calculation in the training procedure. The target dataset is small and comparable to the base training dataset.

Most of these prior existing algorithms for fruit detection and recognition use traditional methods to extract basic features such as color, size, coordinate, and textures by their needs and target images. They have some laminations such as detecting fruits from images with different backgrounds. The major contributions in this paper can be highlighted as follows:

1. We have proposed a simple and efficient deep learning framework for automatic fruit and vegetable recognition
2. Another contribution in this paper is that we established a fruit images database having 20 different categories comprising of 10,000 images
3. The proposed method achieved an accuracy of 96% which implies that the proposed method can be used for real-world applications such as in fruits' shops and supermarkets

## 2. Methodology

In this study, we proposed a simple and efficient fruit and vegetable detection and classification algorithm using a deep convolutional neural network. The main aim of this paper is to apply deep learning with the data expansion techniques to 20 different categories of fruits and vegetables. Deep neural networks take input without any preprocessing. We proposed a simple and efficient machine learning framework with only three convolutional layers and two fully connected layers. The proposed method could learn the best features from a large input image dataset without any preprocessing. An image dataset with various real-world scenarios has been used to test the proposed network's performance. According to the experimental results, the proposed method achieved a high accuracy rate. The detailed architecture of the proposed method has been discussed in Section 2.1.

### 2.1. Proposed Deep Learning Framework Architecture

The proposed deep learning model depends on neural networks. The main advantage of neural network-based models is to learn the events and make decisions by observing similar events [19, 20]. A convolutional neural network model is a kind of neural networks models. However, selecting a convolutional neural network framework for a particular task is not an easy job. In our proposed framework, the first stage is consisting of a deep convolution neural network with max-pooling. The split-and-merge algorithm is used to remove the background of each image.

**Figure 1:**
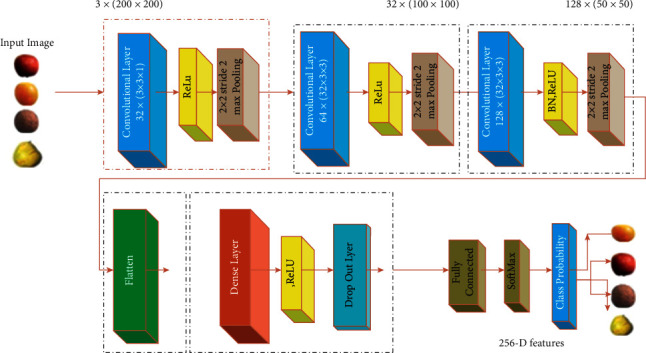
Proposed deep learning framework architecture of fruit classification.

We used true-color images of size 200 × 200 as input to the model. The images are initially in RGB format; then, we converted the images into grayscale. Afterward, we feed the images to the model for further processing. Convolution pooling layer 1 extracts 32 feature maps with a 3 × 3 local receptive field (convolution kernel) and a stride length of 1 pixel, followed by a max-pooling operation in a 2 × 2 region in our proposed model. The convolution layers 2 and 3 also use a  3 × 3 convolution kernel, resulting in 256 feature matrices, and all other parameters remain unchanged. In our network, we used the ReLU activation function because it trained the neural network several times faster without significant plenty to generalization accuracy. After several convolutional and max-pooling layers, we used a fully connected layer. SoftMax neurons correspond to the 20 various categories of fruits and vegetables. The main operations in the proposed network are shown in Figure 1.

Every ConvNet is built on the foundation of convolution, pooling, sampling, and classification, so understanding these processes is critical to developing a solid understanding of ConvNets. Each of these actions will be explained in detail below.

### 2.2. Convolutional Layer

**Figure 2:**
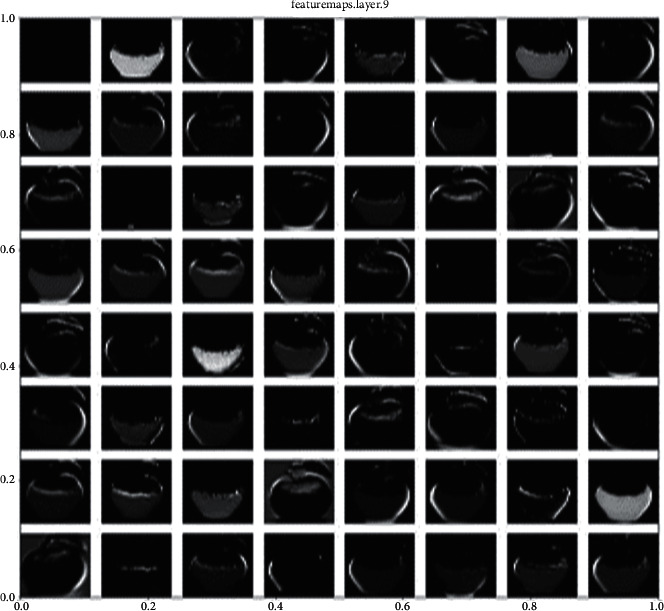
Output feature maps of the convolutional layer.

The convolution function in ConvNet extracts features from the input image and produces feature maps at the output. As a basis for our proposed model, we used three convolutional layers with a 3 × 3 filer. A 5 × 5 convolutional kernel was also tested, but the best results were achieved with a 3 × 3 convolutional kernel. The features maps are produced at the output. The output feature maps' layer 1 is as shown in Figure 2.

### 2.3. Rectified Linear Unit (ReLU)

We used ReLU as an activation function in our proposed deep learning framework. The main purpose of using ReLU as activation is to introduce nonlinearity and also reduce the vanishing gradient problem and accept models to learn faster and perform better.

### 2.4. Pooling Layer

A 2 × 2 window with an average pooling size of 2 × 2 was used and the highest value from the corrected feature map was taken. It is possible to reduce the number of nodes in a network by using the average pooling layer. It is well known that the average pooling reduces the number of parameters and expands the relevant fields.

### 2.5. Fully Connected Layer

The convolutional module in the proposed network transforms the input feature maps into a 128−dimensional feature vector. In the proposed network, we used two layers that were fully connected. Fully connected and Softmax layers are used to construct the linear classification module that is located at the network's output.

### 2.6. Dropout Layer

In the proposed method, we used one dropout layer at the rate of 0.3. The purpose of using the dropout layer is to avoid the model overfitting problem, which may often happen in deep and machine learning models when a function is too closely aligned to a limited set of data points.

## 3. Data Collection

The database used in this analysis contains 10,000 images of fruits taken over two months. All of the images were taken with a resolution of 200 × 200 pixels with an HD Logitech web camera. We encountered a variety of challenges when collecting this database, including light, darkness, sunshine, pose variation, lighting changes, the camera capturing artifacts, and shadows. The split-and-merge algorithm is used to remove the background of each image. To make our model robust, we need to deal with illumination variations, capture artifacts, specular reflection shading, and shadows in real-world recognition scenarios in supermarkets and fruit shops. In all cases, we checked the robustness of our model, and it performed admirably. It was saved in RGB color space, 8-bit per channel. Images for the same category were taken at various times and days. This enhances the dataset's uncertainty and makes the scenario more realistic. There was a lot of variety in the quality and lighting of the images. The fruit data were gathered in a reasonably unrestricted setting. There are also images taken by moving the weight machine near to the windows and then capturing the images by opening and closing the window curtains. The individual number of training samples that we have used to train our proposed model are listed in Table 1.

Data samples with different environmental variations are shown in Table 2. Images of the same group were taken in a variety of settings, including day and night. The classifier's recognition accuracy is hampered by the fact that some fruits have the same color and size. A large amount of data is required to fully understand a deep learning algorithm. When the dataset is small, deep learning algorithms do not work well. A convolutional neural network can be trained to recognize fruits using the data we currently have.

## 4. Simulation Results

**Figure 3:**
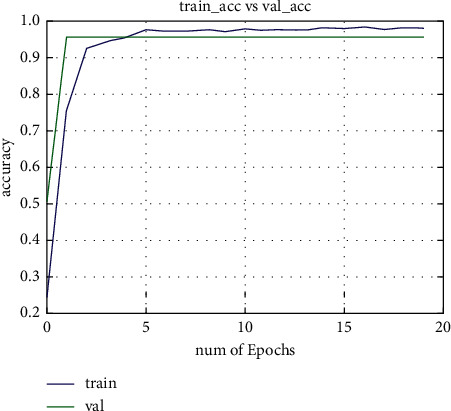
Train and valid accuracy curves.

**Figure 4:**
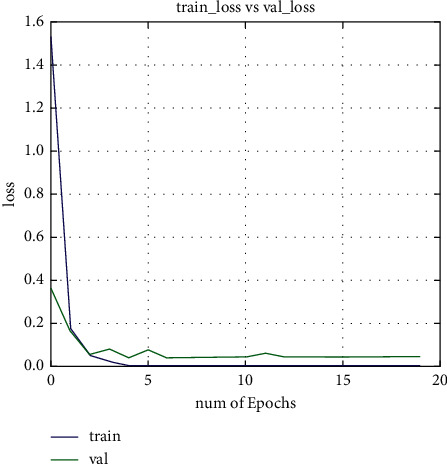
Train and valid loss curves.

Extensive experiments have been carried out to evaluate the proposed network performance on different scenarios such as lightning and pose variations' challenges. As we know that choosing a CNN architecture for real-time object identification and recognition is a tough undertaking because the exact number of layers, kind of layers, and the number of neurons to utilize in each layer are all difficult to determine, in this paper, we have examined different network architectures to find the best one. During the network training, we set the number of epochs to 20. As shown in training and validation loss curves, the training loss is decreasing by increasing the number of epochs, as shown in Figures 3 and 4. After repeating a few trials with different overfitting strategies such as adding dropout layers, using data augmentation, using architectures that generalize well, and adjusting hyperparameters, two significant improvements were obtained. As demonstrated in Figures 3 and 4, the first test accuracy was considerably improved to 96 percent with 20 epochs, and the overfitting issue was eliminated.

## 5. Confusion Matrix

**Figure 5:**
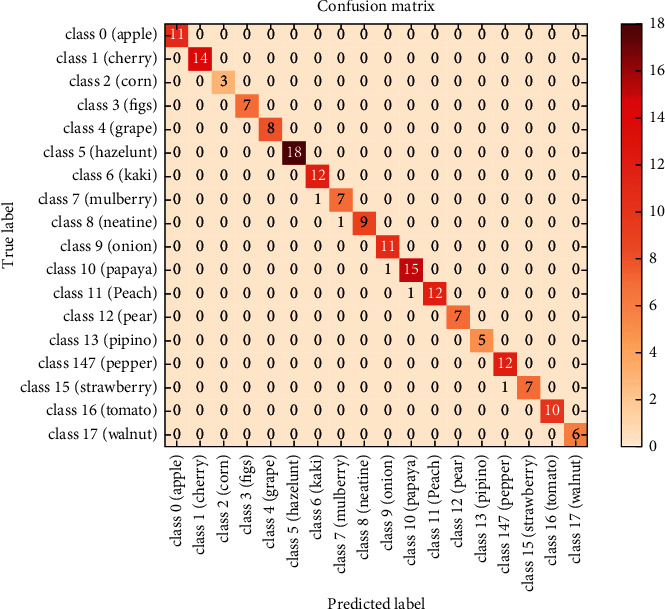
Confusion matrix.

A confusion matrix, as we all know, is an M × N matrix used to analyze the network performance of a classification model, with *N* being the number of output classes. This provides us with a comprehensive picture of how well our classification framework is performing and the types of errors it is making. The matrix compares the actual target values with those predicted by our proposed deep learning framework. In our uncertainty matrix, *X*-axis shows the fruit labels while the *Y*-axis shows the actual labels for fruits, whereas the diagonal element shows the right prediction by the proposed model. The higher the diagonal values of the uncertainty matrix, the higher the right prediction made by our proposed model.  Figure 5 shows the confusion matrix of the model classification results in which the diagonal element shows the correct predictions.

The recognition probabilities for the test images, as well as the respective recognition rates for each fruit category, are shown in our classification report in Table 3. The precision is calculated by dividing the number of true positives by the number of false positives, where TP and FP are the number of true positives and false positives, respectively. Recall essentially tells us how many of the actual positive cases our proposed model were able to predict correctly. The ability of the classifier to locate all positive samples is referred to as recall. The number of times a class appears in a game is referred to as support.

### 5.1. Precision

The precision is also known as the positive predictive value. Precision is the number of positive class predictions that belong to the positive class:

(1) $$\begin{array}{c} \text{Precision}=\left(\text{TP}\right)/\left(\text{Total samples that were predicted as positives}\right), \end{array}$$

where Precision = 0.96.

### 5.2. Recall

Precision and recall are two numbers that are combined to assess a categorization or information retrieval system's performance. The fraction of retrieved instances among all relevant instances is known as recall, also known as sensitivity:

(2) $$\begin{array}{c} \text{Recall}=\left(\text{TP}\right)/\left(\text{Total samples that were positive}\right),\\ \text{Recall}=0.96. \end{array}$$

### 5.3. F1-Score

It is also referred to as an F-score or an F-measure. Specifically, the F1-score reflects the appropriate balance between precision and recall:

(3) $$\begin{array}{c} \text{ F}1-\text{Score}=2*\left(\text{precision }*\text{ recall}\right)/\left(\text{precision}+\text{recall}\right)\\ =0.95. \end{array}$$

## 6. Comparison with Other State-of-the-Art Methods

We compared our results to recent deep neural network-based methods. Hussain et al. [13, 14] proposed some cutting-edge algorithms. The proposed algorithms achieved an accuracy of 99% to recognize the fruits. However, these proposed methods failed to detect fruit images with different backgrounds. Because the authors utilized images with background during training the model and they did not use morphological techniques to remove the background, so the model failed to detect images with different backgrounds. Our proposed method can detect and recognize fruit images with different backgrounds with numerous lighting conditions. The proposed method achieved an accuracy of 96% for 20 different categories of fruits.

## 7. Conclusion

In this paper, we proposed a simple and efficient machine and deep learning-based framework for detecting and recognizing fruits in challenging environments such as lighting and background variations. In a variety of scenarios, the proposed approach was able to recognize fruits images with ease. We included all real-world challenges in our dataset to increase the robustness of the proposed method. As a result, our proposed method significantly improved the identification rate and may be suitable for real-world applications. We tested the network's performance on our image dataset and found that it had a detection accuracy of 96 percent. We compared our results to those of several recently proposed deep learning-based algorithms and discovered that our proposed method outperforms the prior existing methods in a variety of environmental challenges. One of the limitations of the proposed algorithm is that the proposed method does not perform well in the scenario where the model is trained on one dataset and then tested on another dataset. In our future work, we want to expand our dataset and include a greater number of fruit and vegetable categories and also want to investigate the problem of source-target domain mismatch.

## Figures and Tables

**Table 1:** List of images for different fruits.

S#	Fruit name	Fruit image	S#	Fruit name	Fruit images
1	Apple	500	11	Nectarine	500
2	Cherry	500	12	Onion red	500
3	Walnut	500	13	Peach	500
4	Pear red	500	14	Pepper green	500
5	Strawberry	500	15	Papaya	500
6	Grape white	500	16	Pepino	500
7	Fig	500	17	Corn	500
8	Mulberry	500	18	Hazelnut	500
9	Guava	500	19	Kaki	500
10	Apricot	500	20	Grape black	500
Total images for 20 categories is 10,000					

**Table 2:** Example of some training samples of fruits for 20 different categories.

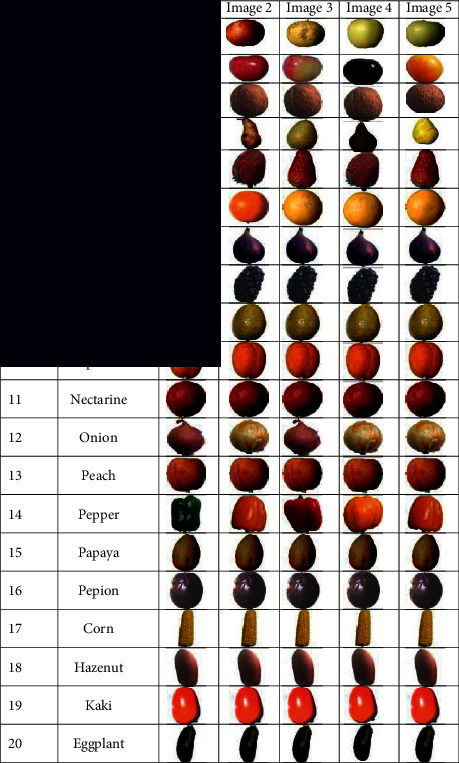

**Table 3:** Evaluation results of fruit classification experiment.

S#	Fruit name	Precision	Recall	F1-score	Support
1	Apple	1.00	1.00	1.00	3
2	Cherry	1.00	1.00	1.00	9
3	Corn	1.00	1.00	1.00	1
4	Fig	1.00	1.00	1.00	3
5	Grape	1.00	1.00	1.00	1
6	Hazelnut	1.00	1.00	1.00	11
7	Kaki	0.88	1.00	0.93	7
8	Mulberry	1.00	0.75	0.86	4
9	Nectarine	1.00	1.00	1.00	6
10	Onion	0.88	1.00	0.93	7
11	Papaya	0.92	0.92	0.92	12
12	Peach	1.00	0.83	0.91	6
13	Pear	1.00	1.00	1.00	1
14	Pepino	1.00	1.00	1.00	3
15	Pepper	0.83	1.00	0.91	5
16	Strawberry	1.00	0.75	0.86	4
17	Tomato	1.00	1.00	1.00	3
18	Walnut	1.00	1.00	1.00	4
**Weighted avg.**		**0.96**	**0.96**	**0.95**	**90**

## Data Availability

The data used to support the findings of this study are available from the corresponding author upon request.
